# Visual Data Exploration for Balance Quantification in Real-Time During Exergaming

**DOI:** 10.1371/journal.pone.0170906

**Published:** 2017-01-30

**Authors:** Venustiano Soancatl Aguilar, Jasper J. van de Gronde, Claudine J. C. Lamoth, Mike van Diest, Natasha M. Maurits, Jos B. T. M. Roerdink

**Affiliations:** 1 Johann Bernoulli Institute for Mathematics and Computer Science, University of Groningen, Groningen, The Netherlands; 2 Department of Neurology, University Medical Center Groningen, University of Groningen, Groningen, The Netherlands; 3 Center of Human Movement Sciences, University Medical Center Groningen, University of Groningen, Groningen, The Netherlands; 4 INCAS³, Assen, The Netherlands; Purdue University, UNITED STATES

## Abstract

Unintentional injuries are among the ten leading causes of death in older adults; falls cause 60% of these deaths. Despite their effectiveness to improve balance and reduce the risk of falls, balance training programs have several drawbacks in practice, such as lack of engaging elements, boring exercises, and the effort and cost of travelling, ultimately resulting in low adherence. Exergames, that is, digital games controlled by body movements, have been proposed as an alternative to improve balance. One of the main challenges for exergames is to automatically quantify balance during game-play in order to adapt the game difficulty according to the skills of the player. Here we perform a multidimensional exploratory data analysis, using visualization techniques, to find useful measures for quantifying balance in real-time. First, we visualize exergaming data, derived from 400 force plate recordings of 40 participants from 20 to 79 years and 10 trials per participant, as heat maps and violin plots to get quick insight into the nature of the data. Second, we extract known and new features from the data, such as instantaneous speed, measures of dispersion, turbulence measures derived from speed, and curvature values. Finally, we analyze and visualize these features using several visualizations such as a heat map, overlapping violin plots, a parallel coordinate plot, a projection of the two first principal components, and a scatter plot matrix. Our visualizations and findings suggest that heat maps and violin plots can provide quick insight and directions for further data exploration. The most promising measures to quantify balance in real-time are speed, curvature and a turbulence measure, because these measures show age-related changes in balance performance. The next step is to apply the present techniques to data of whole body movements as recorded by devices such as Kinect.

## Introduction

Incidence of falls commonly cause serious injuries and loss of independence among the older population. In fact, 20–35% of people more than 65 years old fall each year; this number increases to 32–42% for people over 70 years old [1]. Approximately 20–30% of those people will experience a lack of mobility and independence, thus increasing the risk of death [2, 3]. Furthermore, unintentional injuries are among the ten leading causes of death in older adults and falls cause 60% of these deaths [4]. Although there are many factors that contribute to falls, poor balance is one of the major risk factors for falling due to the natural age-related decline of sensory and neuromuscular control mechanisms that result in impaired postural control [5]. Balance training programs can improve balance ability, thereby reducing the risk of falls and injuries [6]. However, such programs have not been as successful as expected because of several drawbacks, like lack of motivating elements, the effort and cost of travelling, or boring exercises, ultimately resulting in low adherence [7, 8].

Given the great popularity of digital games around the world at all ages, exergames have been proposed as an alternative to improve balance among older adults [9–12]. Exergames are digital games controlled by real-time body movements recorded with tracking technology such as inertial measurement units, infrared cameras, and force plates [11, 13]. The most common methods to study the effectiveness of exergames, based on balance improvement, rely on assessing balance before and after exergame training [10]. However, balance control is typically not assessed *during* gameplay (in real-time). This kind of assessment could be used to adjust the exergame difficulty level according to the performance and skills of each individual player. In addition, appropriate adaptive feedback can be provided based on real-time performance. Furthermore, appropriate feedback can increase motivation to play and therefore improve effectiveness and adherence of exergames [14, 15].

The main goal of this study is to conduct an exploratory multidimensional data analysis, deriving metrics from exergame data recordings and using visualization techniques, to establish measures that can be used to quantify balance ability in real time during exergaming.

**Fig 1:**
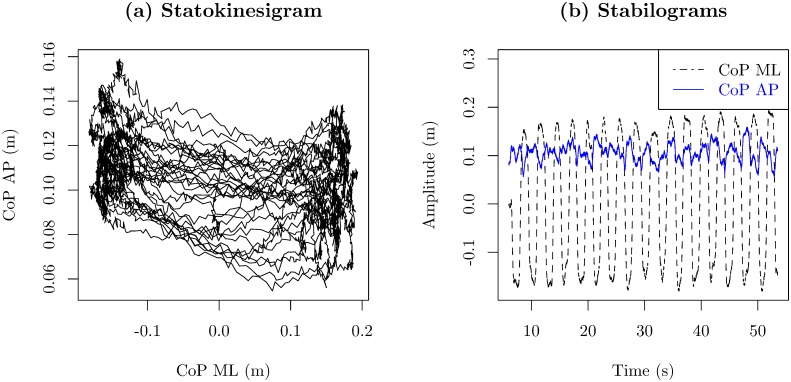
Typical ways of visualizing trajectories of the CoP. (a) statokinesigram and (b) AP and ML stabilograms.

Balance control or postural control is defined as the ability to maintain the center of body mass (CoM) within limits of stability determined mostly by the base of support (the feet) during static or dynamic tasks [16, 17]. When the CoM falls out of the base of support, humans have the ability to use muscular reaction against the force of gravity to prevent falling, i.e., postural control. One of the most common ways to quantify balance is by extracting measures derived from force plate recordings. A force plate is a device that measures three-dimensional ground reaction forces, consisting of an anterior-posterior (AP), a medial-lateral (ML), and a vertical component [18]. These forces are used to derive the center of pressure (CoP) trajectories, in AP and ML directions. The CoP is the location of the vertical ground reaction force vector [19]. CoP trajectories are commonly visualized by a statokinesigram or a stabilogram. The statokinesigram is a plot of the AP direction versus the ML direction (Fig 1(a)), while the stabilogram is a plot of the individual CoP AP and ML time series (Fig 1(b)) [20]. Although these kinds of visualizations are good enough to examine one or two CoP time series, they are not appropriate for visualizing multiple CoP time series because plots will be too cluttered and unintelligible. The ability to simultaneously visualize multiple CoP trajectories could unveil hidden balance control patterns providing insight for further data exploration.

Quantification of balance changes by means of force plates is typically done by using average scalar parameters derived from CoP trajectories, such as mean velocity and total distance; or measures of dispersion around the mean, like root mean square (RMS), standard deviation (SD), and coefficient of variation (CoV) [21, 22]. Two of the strongest limitations of these measures are: (1) averaging suppresses information relevant for understanding the time-varying structure of postural sway patterns across time; and (2) establishing the validity of the measurements is not possible because there is neither an ideal nor a perfect CoP trajectory (gold standard) [23]. Thus, although typical measures have been used for successfully quantifying balance during static tasks, they are not suitable for studying the temporal dynamics of the CoP [23] and cannot be used for real-time balance quantification in dynamic tasks because these measures depend on the whole trajectory.

Continuous methods provide an alternative for quantifying CoP trajectory variability as a function of time. Measures derived from the theory of stochastic dynamics have been employed to quantify the time-varying structure of postural sway patterns during both static as well as dynamic tasks [24]. Even during quiet upright standing, an irregular small amplitude body sway is continuously present. An extensive number of studies in the area of motor control have shown that this variability does not only reflect noise but results from a complex interplay of non-linear deterministic and random components [24–27]. Getting insight into this time-varying structure during balance control might provide insight into the underlying mechanisms, and may distinguish healthy from pathological motor control processes [27].

Many methods have been used to study the temporal dynamics of the CoP trajectory, such as recurrence plots, Brownian motion, entropy measures, and Lyapunov exponents [23, 26, 28–30]. Variability of balance control can be considered as a continuum, with normal or healthy variability positioned between two extremes. This view is in line with the notion that health is characterized by ‘organized’ variability, while disease is defined by a loss of complexity, increased regularity, and either increase or decrease of variability, depending on the task to be performed and the patient group [31]. Increased regularity and loss of complexity of postural sway have been reported for several patient groups, including stroke patients [30], athletes with sports-related concussions [23], older adults [5], patients with Parkinson’s disease [32], and children with Cerebral Palsy [33]. In general, such characteristics are deemed to reflect a less efficient and less automatized form of postural control that is less adaptable and more susceptible to external perturbations. However, a drawback of these non-linear methods is that they heavily rely on whole trajectories over long periods of time. This limitation makes these methods inappropriate for assessing balance during game-play when balance needs to be quantified in the order of milliseconds to seconds.

### Methodological approach

The methodological approach taken here is the following: (1) understanding the complexity and nature of CoP trajectories, from recordings of participants from a broad age range, by simultaneously visualizing multiple trajectories; (2) achieving real-time balance quantification by selecting measures that can be estimated for short periods of time (milliseconds), and measures that reflect variability and smoothness of the trajectories such as instantaneous speed, local measures of dispersion from the mean, turbulence measures, and curvature values; (3) analyzing the results by using several visualization techniques.

In the absence of a gold standard and therefore not knowing if a perfect CoP trajectory is desired or can be achieved, we here investigate how age, which is known to influence balance control [5], is related to our extracted CoP features.

## Materials and Methods

For this study we used the data collected in the context of the project *Exergaming for balance training of older adults at home* [34, 35] of the research center SPRINT of the University Medical Center Groningen (UMCG). In this research center a custom-made ice-skating exergame has been developed for unsupervised training of balance of older adults. Additional information about the exergame and SPRINT can be found in [36].

### Participants

For this study forty healthy participants were investigated; 20 older (8 females, 12 males; 71.9 ± 4.0 years) and 20 younger adults (11 females, 9 males; 37 ± 16.6 years). Being physically fit and able to walk for at least 15 minutes without aid (self-reported), and BMI < 30 were considered as inclusion criteria. Musculoskeletal, visual or neurological impairments, or use of medication that could affect postural control, eye or hearing impairments that might affect balance ability or gaming experience, and inability to understand Dutch language, were considered as exclusion criteria. The study involving older adults was performed with the approval of the Medical Ethical Committee, UMCG (approval METc 2013/244), and was executed in accordance with the ethical standards of the declaration of Helsinki. The study involving younger adults was performed with approval of the Ethical Committee of the Center of Human Movement Sciences at the UMCG. All participants signed written informed consent. Further details can be found in [34, 37].

### Procedure and instrumentation

The participants played the exergame for about 50 seconds by swaying the center of body mass in lateral directions in five different conditions: (1) neutral swaying at self-selected speed; (2) speeding up the game by a factor of two; (3) swaying at maximum frequency at a self-selected amplitude; (4) lifting the contra-lateral leg; and (5) swaying at maximum amplitude at a self-selected frequency. All participants performed each trial twice, resulting in 10 trials per participant. In total, 40 × 10 = 400 trials were non-uniformly sampled at a frequency of about 170Hz using force plates. During the trials Kinect and VICON recordings were captured as well and used for different studies [34, 37].

### Data preprocessing

The data were re-sampled at a fixed rate of 170Hz, using cubic spline interpolation in Matlab R2015b, to deal with possible sample frequency deviations. Raw (non-smoothed) data were used for analysis. On average the trials lasted 48 seconds. The first 5-6 seconds were used mostly to prepare the participant for the trial resulting in different types of movements which were not part of the swaying exercise. In some trials, force plate recordings continued up to 6 seconds after the end of the swaying exercise. Therefore, for each trial, the first and last 6 seconds were removed to avoid motions that were not part of the exercise, leaving 36 seconds per trial on average for analysis. Finally, CoP trajectories for each trial were computed as described in [19].

### Measures to quantify balance

Features were extracted using the R language for statistical computing and graphics, R version 3.2.4 Revised (2016-03-16 r70336) [38], the R data.table package version 1.9.6 [39], platform: x86_64-pc-linux-gnu (64-bit) under Ubuntu precise (12.04.5 LTS).

A trajectory is viewed as the path described by a moving point as follows:

$$\gamma \left(t_{i}\right)=\left(ML\left(t_{i}\right),AP\left(t_{i}\right)\right),\;i=1,\ldots ,N$$ (1)

were *γ*(*t*_*i*_) denotes the position vector of the CoP at time *t*_*i*_, *AP* and *ML* are the anterior-posterior and medial-lateral coordinates of the CoP, and *N* represents the number of points on the trajectory.

Fluctuations from the mean (*FM*) were computed as follows:

$$FM\left(t_{i}\right)=\sqrt{{\left(AP\left(t_{i}\right)-\bar{AP}\right)}^{2}+{\left(ML\left(t_{i}\right)-\bar{ML}\right)}^{2}},$$ (2)

where, $\bar{AP}$ and $\bar{ML}$ are the averages of *AP*(*t*_*i*_) and *ML*(*t*_*i*_), and *i* = 1 … *N*. Based on Eq (1) we computed instantaneous speed as follows:

$$v\left(t_{i}\right)=\frac{\|\gamma \left(t_{i}\right)-\gamma \left(t_{i-1}\right)\|}{t_{i}-t_{i-1}},\;i=2,\ldots ,N-1,\;v\left(t_{1}\right)=0,$$ (3)

where *v*(*t*_*i*_) represents the CoP speed at time *t*_*i*_, and ‖.‖ indicates the Euclidean norm.

#### Measures of dispersion

Although traditional measures are not suitable to study the temporal dynamics of the CoP, some of these variables can also be used in a continuous form by integrating a time window into the definition. In this way CoP temporal patterns can also be analyzed for short periods of time. We use the following adapted equations:

$$SD\left(t_{k}\right)=\sqrt{\frac{1}{s}\sum_{i=k-n}^{k+n}{\left(FM\left(t_{i}\right)-\bar{FM_{k}}\right)}^{2}},$$ (4)

and

$$RMS\left(t_{k}\right)=\sqrt{\frac{1}{s}\sum_{i=k-n}^{k+n}{\left(FM\left(t_{i}\right)\right)}^{2}},$$ (5)

where *SD*(*t*_*k*_) is the local standard deviation, $\bar{FM_{k}}$ is the local mean of *FM*(*t*_*i*_) at time *t*_*k*_ within the time window of size *s* equal to 2*n* + 1, *RMS*(*t*_*k*_) is the root mean square at time *t*_*k*_, and *k* = *n* + 1, …, *N* − *n*.

The coefficient of variation (CoV), also known as “coefficient of relative variability”, allows for comparison of data with different central tendencies; it is unitless and scale invariant [40]. Thus, the CoV can provide additional information about the relative dispersion of the data within a particular participant or group. The CoV in its continuous form is defined as the standard deviation normalized by the mean:

$$CoV\left(t_{k}\right)=\frac{SD(t_{k})}{\bar{FM_{k}}}.$$ (6)

#### Variants of measures of dispersion

Measures derived from Eqs (4), (5) and (6) do not take into account distances between points in the CoP trajectory, but only distances from the mean. The former distances should be considered in the calculations, because the measures that include time windows do not take into account that participants could be moving at different speeds, which could result in similar measures of deviation. Higher speeds will produce larger distances travelled between time points and smoother trajectories. To take variable distances between points in the CoP into account, we modified the measures of dispersion, Eqs (4)–(6), by dividing by the distance travelled within the time window, as follows:

$$SD^{\prime }\left(t_{k}\right)=\frac{SD(t_{k})}{d(t_{k})},$$ (7)

$$RMS^{\prime }\left(t_{k}\right)=\frac{RMS(t_{k})}{d(t_{k})},$$ (8)

$$CoV^{\prime }\left(t_{k}\right)=\frac{CoV(t_{k})}{d(t_{k})},$$ (9)

where $d\left(t_{k}\right)=\sum_{i=k-n}^{k+n}\left\|\gamma \left(t_{i+1}\right)-\gamma \left(t_{i}\right)\right\|$ represents the distance travelled along the trajectory within the time window *t*_*k*_, where *k* = *n* + 1, …, *N* − *n*.

#### Turbulence intensity

According to Bradshaw and Woods [41], turbulence is the most complicated kind of fluid motion. Some of the main features of turbulence are spatio-temporal randomness, irregularity, loss of predictability, and high dissipation [42]. Turbulence has been studied for more than a century at all possible scales, from the interior of cells to super-galactic scales. Despite the difficulty of understanding turbulence, turbulence measures usually involve simple properties of motion fluctuation as observed in parameters such as temperature and speed [43]. If we think of balance control as motion fluctuation produced by stabilization of the body during static and dynamic tasks, turbulence measures could be used to characterize balance control. Indeed, a common unitless measure of turbulence intensity is the CoV of speed Eq (10), i.e., the standard deviation of speed, normalized by its mean [43, 44]:

$$I\left(t_{k}\right)=\frac{\sqrt{\frac{1}{s}\sum_{i=k-n}^{k+n}{\left(v\left(t_{i}\right)-\mu \left(t_{k}\right)\right)}^{2}}}{\mu (t_{k})},\;\mu \left(t_{k}\right)=\frac{1}{s}\sum_{i=k-n}^{k+n}v(t_{i})$$ (10)

Here *I*(*t*_*k*_) is the turbulence intensity at time *t*_*k*_, *s* = 2*n* + 1 is the size of the running window, and *k* = *n* + 1, …, *N* − *n*. We also defined a variant of Eq (10) by using the mean square instead of the mean as denominator:

$$I^{\prime }\left(t_{k}\right)=\frac{\sqrt{\frac{1}{s}\sum_{i=k-n}^{k+n}{\left(v\left(t_{i}\right)-\mu \left(t_{k}\right)\right)}^{2}}}{\mu^{\prime }\left(t_{k}\right)},\;\mu^{\prime }\left(t_{k}\right)=\frac{1}{s}\sum_{i=k-n}^{k+n}v{\left(t_{i}\right)}^{2}$$ (11)

#### Curvature

**Fig 2:**
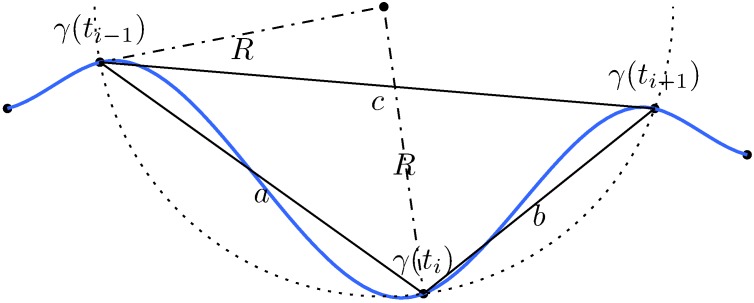
Schematic representing the elements used to approximate curvature values. The blue curved line represents the trajectory, *γ*(*t*_*i*−1…*i*+1_) are the involved points to estimate the curvature value at the *γ*(*t*_*i*_) point, *a*, *b* and *c* are the sides of the triangle, the dotted line is the fitted circle, and *R* is its radius.

Curvature measures the degree to which a curve is not a straight line. A straight line has zero curvature, and large circles have smaller curvature than small circles [45]. In this sense, more fluctuating or irregular trajectories should have larger curvature values. Thus, curvature may be useful to further characterize CoP trajectories. Curvature values along a trajectory can be approximated by the curvature of a circle passing through three consecutive points [46] as follows:

$$\kappa \left(t_{i}\right)=4\frac{{△}_{abc}}{abc}=4\frac{\sqrt{\hat{s}\left(\hat{s}-a\right)\left(\hat{s}-b\right)\left(\hat{s}-c\right)}}{abc},$$ (12)

where *a* = ‖*γ*(*t*_*i*_) − *γ*(*t*_*i*−1_)‖, *b* = ‖*γ*(*t*_*i*+1_) − *γ*(*t*_*i*_)‖ and *c* = ‖*γ*(*t*_*i*+1_) − *γ*(*t*_*i*−1_)‖ (see Fig 2), △_*abc*_ is the area of the triangle defined by the points *a*, *b*, *c*; $\hat{s}=\left(a+b+c\right)/2$ is half of the triangle perimeter (from Heron’s formula [47]), and *i* = 2, …, *N* − 1.

### Balance measure extraction

To select the window size *s* in Eqs (4)–(11) we considered, as the main constraint, *s* to be shorter than half of a sway cycle, i.e., shorter than a CoP transition between feet, as otherwise some temporal details might be missed. The maximum sway frequency among older and younger participants for this particular experiment is about 0.65Hz [34]. Thus, we tested several window sizes within the range of 0.2 to 0.5 seconds, which yielded similar results. Here we show results using a window size of 0.3 seconds. As the CoP trajectories were re-sampled at 170Hz, we used *n* = 25 and *s* = 2*n* + 1 = 51 samples.

After computing the local measures for each trajectory described by Eqs (3)–(12), the medians per trajectory were extracted. Note that for curvature values we do use both means (denoted by $\bar{\kappa }$) as well as medians because these measures do not seem to be as sensitive to outliers as the other measures. A reason might be that for points on the trajectory far away from the force plate area, curvature values are small compared to those within the force plate area, thus having a small effect on the mean. Medians and means were stored in a matrix of 400 rows (trials) by 11 columns (measures), available as Supporting Information.

### Statistical analyses

To investigate whether balance measures for older and younger participants were significantly different the following tests were performed. The Shapiro-Wilk test [48] was used to determine whether balance measures in each group followed normal distributions. If this was indeed the case, balance measures were compared between groups using T-tests, otherwise the Mann-Whitney U-test was used [49]. Bonferroni correction was applied to correct for multiple comparisons.

## Results

### Multiple CoP trajectory visualization

The main purpose of the visualizations in this subsection is to gain quick insight into the structure of the CoP trajectories with the least possible preprocessing. To achieve this, we visualized the CoP ML movement using heat maps and violin plots.

#### Heat map

One of the most space-efficient ways to visualize data is a pixel-based representation [50]. An instance of this kind is the heat map, which is typically a rectangular tiling of a color-shaded data matrix. Heat maps allow for the simultaneous exploration of several thousands of rows and columns [51]. Inspired by heat maps, we plotted CoP ML trajectories as ordered scatterplots. Each point is color-shaded as a function of CoP ML position and plotted at coordinate (*it*, *t*), where *t* represents time on the vertical axis, *it* is an index along the horizontal axis used to represent each CoP ML trajectory as a vertical line, *it* = (*np* − 1) × 11 + *trial* = [1, …, 440], where *np* is the index of participant [1, …, 40] (ordered by age), 11 is the number of trials per participant (the 11*th* trial is an empty one used to separate each ten trials per participant), and *trial* is the index of trial [1, …, 11].

Force plate recordings may contain erroneous measurements, resulting in values extremely far away from the average and outside of the force plate area (outliers). Color-shading functions are very sensitive to outliers because they cause most of the values to be projected into a small section of the color range, thereby hiding the main structure of the data. According to the experimental set up [34], participants were asked to keep their feet within an 80 × 60 cm^2^ area. Thus, to avoid the effect of outliers, values outside of this area were excluded from plotting. As the number of outliers is limited (< 0.05%) and the sampling rate is high (170Hz), the difference is unnoticeable.

**Fig 3:**
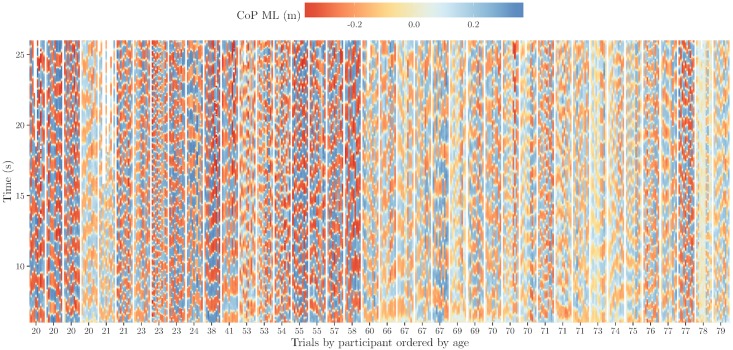
Heat map visualizing 400 CoP ML stabilograms. The horizontal axis represents trials per participant, with participants ordered by age. The vertical axis represents time from 6 to 26 seconds. The color-shaded vertical lines represent the CoP medial lateral position per trial.

Fig 3 shows the 400 CoP ML stabilograms during 20 seconds as a heat map. This figure reveals several interesting features:

1. In general, younger participants (20–60 years old) have larger CoP ML amplitudes than older participants, as indicated by the higher color intensity in the younger participants. This visualization is consistent with studies reporting physical decline particularly after 60 years of age. For example, a recent study [52] reports evident decline in walking speed and aerobic endurance for people in their 60s and 70s;
2. Younger participants move at higher speeds than older participants, as can be observed from the higher frequency of the vertical transitions in younger participants;
3. Sharp and clear transitions indicate that CoP trajectories among younger participants are smoother than among older participants;
4. Other particular observations are: some of the trials recorded from the first participant aged 21 did not last at least 20 seconds, the second participant aged 23 seems to be the fastest, and the second participant aged 77 shows the largest amplitude and the most clear CoP ML transitions among older participants. Indeed, this participant seems to behave as a young participant.

#### Violin plots

**Fig 4:**
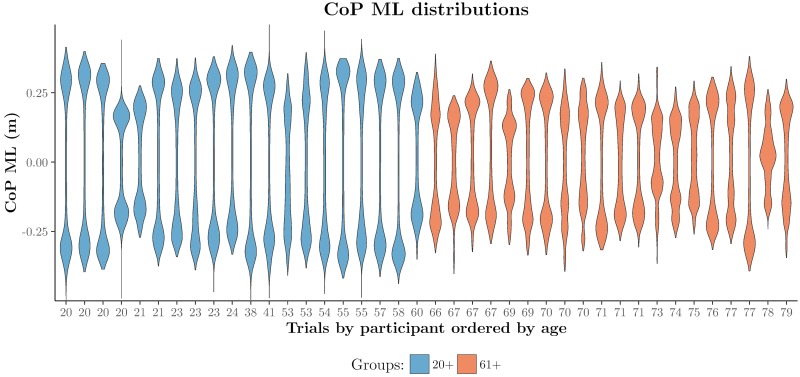
Violin plots representing CoP ML transitions between feet. The horizontal axis represents the distribution of CoP ML measurements per participant as violin plots, and the vertical axis represents the CoP ML coordinate. This figure appeared in [54]. Eurographics Proceedings 2016. Reproduced by kind permission of the Eurographics Association.

One of the main strengths of violin plots is their potential to reveal peaks, valleys, and bumps in the shape of distributions [53]. These features could be useful for the identification of clusters and for comparison of distributions. Fig 4 shows violin plots of the CoP ML trajectories of the ten trials per participant, ordered by age. These plots include data of complete CoP trajectories (not only 20 seconds) and are displayed along the horizontal axis. The vertical axis represents the CoP ML coordinate. In a different way, this figure confirms several observations made for Fig 3. For example, CoP ML amplitudes are clearly larger among younger participants, as can be derived from the distances between the bumps (higher densities) at the extremes of the distributions. Because the data were re-sampled at 170Hz, higher densities indicate more samples and therefore more time than lower densities. Thus, bumps also indicate that most of the time the CoP is under one of the feet. The lower densities of samples (valleys) indicate higher speeds among younger than older participants. Indeed, if we look carefully between “red” and “blue” transitions in the heat map, there is a smaller “yellow” line, the length of this line representing the time of the transition. This is easier to observe in the violin plots than in the heat map by looking at the thickness of the distributions between bumps. The violin plots also clearly show a participant that behaves differently from the other participants, the 78 year old participant who keeps the CoP between his/her feet most of the time, as illustrated by the bump in the middle of the distribution (completely opposite to the rest of the distributions), indicating a low degree of sway in the ML direction. In fact, if we look back at Fig 3 and closely look at this participant, we can see that in trials 3–6 there are almost no CoP ML transitions. This observation explains the shape of the distribution.

In summary, heat maps and violin plots together allow to gain insight into speed, frequency, amplitude, and smoothness of the CoP trajectories. We can also see differences between participants and types of trials. In addition, based on the above observations we expect higher measures of variability (SD, Eq (4), and RMS, Eq (5)) in younger than older participants because of their higher CoP ML sway amplitudes. We also expect higher curvature values among older than younger participants, as indicated by the smoother trajectories among younger participants. Naturally, higher speeds are expected for younger than for older participants as well.

### Visualization of multiple features

In this subsection we use a heat map, violin plots, a parallel coordinate plot, and a scatterplot matrix to visualize our results, because these types of visualizations are some of the appropriate techniques to explore multidimensional data [50, 55]. Each visualization leads to different insights into the behavior of the participants allowing us to identify the best measures for real-time quantification of balance.

#### Heat map

**Fig 5:**
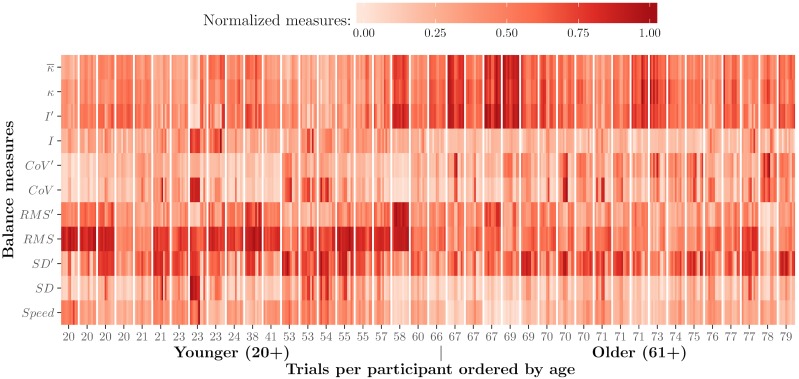
Heat map visualization of measures by trial and participant. The horizontal axis represents trials, as vertical lines, per participant, with participants ordered by age; the vertical axis represents our measures of balance ($\bar{\kappa }$—mean curvature, *κ*—median curvature, “′” represents a variant of the measure, *I*—turbulence intensity, *CoV* coefficient of variation, *RMS*—root mean square, *SD*—standard deviation), and shades of “red” indicate higher and lower values for measures of balance. This figure appeared in [54]. Eurographics Proceedings 2016. Reproduced by kind permission of the Eurographics Association.

Taking advantage of heat map features we simultaneously visualized and analyzed trials, participants, and measures. Fig 5 shows the 400 × 11 matrix normalized and visualized as a heatmap. The horizontal axis represents trials of participants, ordered by age. The vertical axis represents the different measures used in our calculations. Each measure per trajectory is represented as a color-shaded vertical line. Darker colors represent higher values than lighter colors. Ten consecutive vertical lines form a bar which represents ten trials per participant.

In Fig 5, values of curvature *κ* and turbulence intensity *I*′ are lower for younger participants than for older participants, and speed is slightly higher for younger participants. The measure that shows the most clear differences between younger and older participants is *RMS*. The rest of the measures are not clearly different between older and younger participants. In this figure and for this particular sample, it is also possible to identify differences within groups of participants. For example, participants from 58 to 69 years old seem to have the most irregular trajectories, as darker curvature colors indicate more irregular trajectories than lighter colors. These participants are also the slowest ones in the group, as can be derived from speed values in the same columns. The fastest participants in our group are from 53 to 57 years old, who scored low curvature values.

#### Overlapping violin plots

Fig 5 provides qualitative insight into balance measures showing differences between younger and older participants. What is missing, however, is a quantitative measure of these differences. We use the overlapping coefficient (OVL), defined as the area of overlap between two probability density functions [56], as such a measure. If *f*_1_(**x**) and *f*_2_(**x**) represent the younger and older density curves, respectively, the OVL can be determined as follows:

$$\text{OVL}=\sum_{x}min\left[f_{1}\left(x\right),f_{2}\left(x\right)\right].$$ (13)

**Fig 6:**
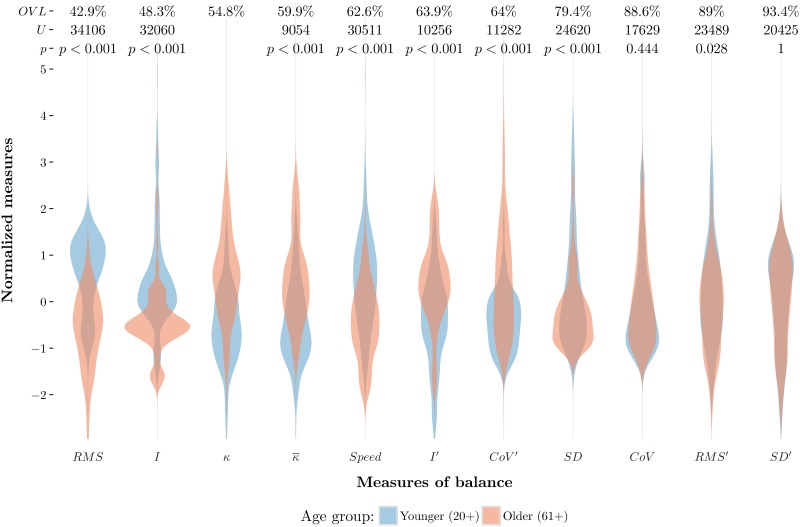
Overlapping violin plots showing differences between older and younger participants. The three values above each pair of overlapping violin plots are the overlapping area (OVL), the U-statistic and the p-values. The t-test results for *κ* are in the main text.

The OVL has the following properties: (1) 0 ≤ OVL ≤ 1, (2) OVL = 0 if and only if there is no overlap area between the two curves, and (3) OVL = 1 if and only if the two curves are identical [56, 57]. Thus, the OVL provides a quantitative measure of the difference between older and younger participants. Fig 6 shows along the *x*-axis the 11 measures and their density curves, as violin plots, grouped by older and younger participants. Above each violin plot there are three values, the OVL, the *U*-statistic and the *p*-value, the latter two resulting from statistical comparisons. The violin plots are sorted according to the OVL between groups. The *y*-axis represents the normalized measures. To normalize the data, for each value the mean was subtracted and the result was divided by the standard deviation.

The Shapiro-Wilk test indicated that only *κ* was normally distributed (*W* > 0.99, *p* > 0.05) for both older and younger groups. Mean *κ* (182.09) for the older participants was significantly lower than the mean *κ* (239.4) for younger participants (*t* = −12.21, *df* = 387.25, *p* < 0.01). Younger and older participants did not differ for *CoV* or *SD* measures (*p* > 0.05). Younger and older participants differed on all other measures (see Fig 6).

Fig 6 allows to gain additional information about the measures and their potential to differentiate between groups of participants. *SD*′, *RMS*′, *CoV* and *SD* show the smallest differences between groups (OVL > 79%); *CoV*′, *I*′ and *Speed* show better distinction with an overlap between 62.6% and 64%. Consistent with the heat map visualization (Fig 5), the violin plots show that *RMS* and *κ* are measures that show clear differences between groups. Furthermore, Fig 6 reveals that *I* should also be considered for further exploration, because this measure shows the second lowest OVL among the measures.

#### Parallel coordinates

Parallel coordinate plots (PCP) provide a practical way to visualize multivariate data. This kind of visualization has a number of features that are desirable for a good visualization of multivariate data, like low representational complexity, invariance to rotation, translation and scaling, mathematical rigour, and ease of data exploration [58]. Despite of the popularity of PCP within the visualization community, they are still unknown in many other domains [59]. Balance quantification is an example of such a domain.

One of the main drawbacks of PCP is the visual clutter that results from the overlap of too many polylines hiding the main structure of the data [60]. A common way to minimize visual clutter is by rearranging the parallel axes [61]. According to [59], there are several metrics to reorder the parallel axes such as measuring Euclidean distance, overlap, and number of line crossings. To reduce visual clutter we reordered the axes using the same order as in Fig 6, from minimum to maximum overlap between older and younger groups.

**Fig 7:**
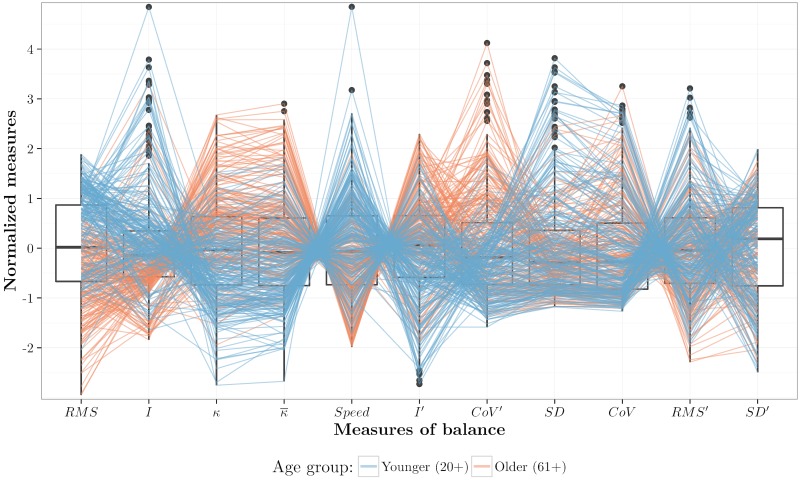
Normalized measures as a parallel coordinate plot. In the box plots points beyond the whiskers are outliers as specified by [62].

In Fig 7, the parallel axes represent the measures derived from Eqs (3)–(12). Behind each axis is a boxplot, where points located outside the boxplot whiskers represent outliers: values beyond 1.5 times the corresponding interquartile range [62]. Each color-shaded-transparent polyline represents the values derived from one trajectory. In addition to the previous observations, this visualization highlights the following:

1. the parallel axes and the transparent polylines together provide a different view of the overlap between groups;
2. *I*, *CoV*′, and *SD* are the measures most sensitive to outliers, as indicated by the boxplots;
3. clusters of polylines suggest strong correlations between some measures. For example, trajectories with high *I*-values are mapped onto low *κ*-values and vice-versa, something similar happens between $\bar{\kappa }$, *Speed* and *I*′.

#### Projections

**Fig 8:**
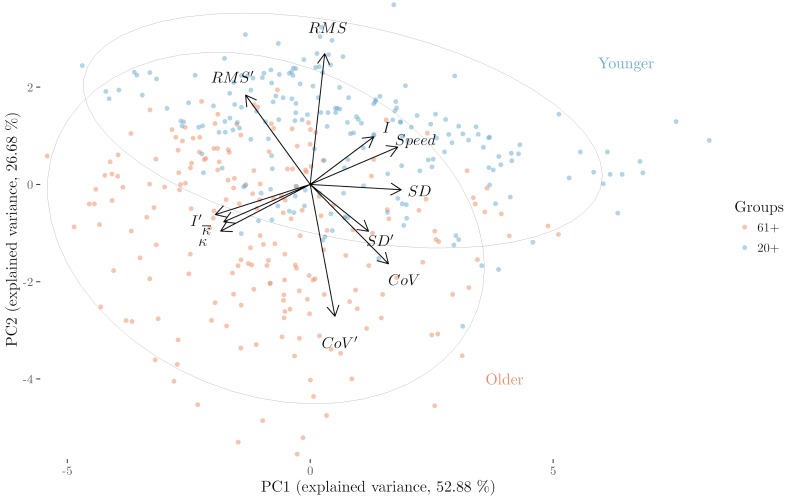
Biplot and projection of the balance measures onto the first two principal components PC1 and PC2. The arrows represent the variable vectors and the ellipses cluster older and younger participants. This figure was adapted from [54]. Eurographics Proceedings 2016. Reproduced by kind permission of the Eurographics Association.

Projections are particularly useful to gain additional insight when the data lie close to a two- or three-dimensional subspace [63]. We applied principal component analysis (PCA) to investigate whether this is the case and how we could visualize groups. PCA showed that the first four principal components (PCs) account for more than 95% of the variance and the first two PCs account for more than 79% of the variance. Fig 8 shows a biplot with vectors labeled with variable names at their end-points. The end-points are the projections of the eigenvectors corresponding to the first two PCs. For better visualization the vectors were multiplied by 5, as multiplying the eigenvectors by a constant does not change the biplot interpretation (see [64], p. 403). In addition, we used the first two PCs to project data points onto a two-dimensional subspace. In this plot each color-shaded point represents a trajectory. Color represents older and younger participants. Two 95% confidence ellipses were drawn, based on [65], to better visualize older and younger clusters.

Fig 8 illustrates that the magnitudes of *RMS*, *CoV*, and their variant vectors are larger than the rest of the variables indicating stronger contribution to the PCs. In addition, their directions (more aligned to the vertical axis) indicate stronger contribution to the second PC. The angles between the variable vectors can provide additional information about the correlation between variables [66]. Orthogonal or almost orthogonal vectors indicate weak correlation, opposite vector directions indicate strong negative correlations, and similar directions indicate strong positive correlations. Thus, *CoV* and *CoV*′ are negatively correlated with *RMS* and *RMS*′; *SD* is negatively correlated with *κ* and *I*′; *Speed* and *I* are strongly negatively correlated with *κ* and *I*′, while the rest of the correlations seem to be weak. The ellipses clearly show how younger participants are grouped together in the upper part of the projection (upper ellipse), while trials from older participants are dispersed in a larger area in the lower part of the projection (lower ellipse), having some overlap with younger participants. This overlap suggests that some older participants behave like younger participants and vice-versa. Fig 8 also illustrates that the second principal component accounts for most of the variability that may represent age-related differences among participants, as the two groups are most strongly separated along this axis.

**Fig 9:**
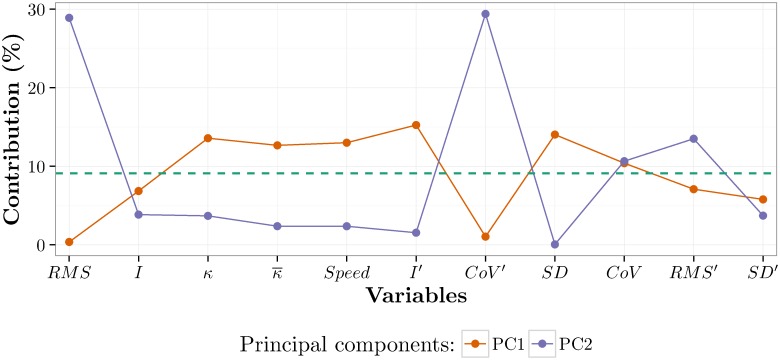
Contribution of the variables to the first two PCs. The horizontal axis represents the variables and the vertical axis represents the percentage of contribution. The green dashed line represents the mean contribution of the variables (100/11).

To estimate the contribution of each variable as percentages to the first two PCs, the squared loadings between the variables and the PCs were multiplied by 100, as the square loadings reflect the contribution of the variables to the PCs [67]. In this manner, the variables that account for most of the variability in the PCs can be identified. Fig 9 shows the contributions of the variables to the first two PCs, illustrating that *RMS* and *CoV*′ contribute most to PC2 and therefore probably account for most of the age-related differences visible in Fig 8. Similarly, *κ* values, *Speed*, *I*′, *SD* and *CoV* are the most relevant variables for PC1.

#### Scatter plot and correlation matrix

**Fig 10:**
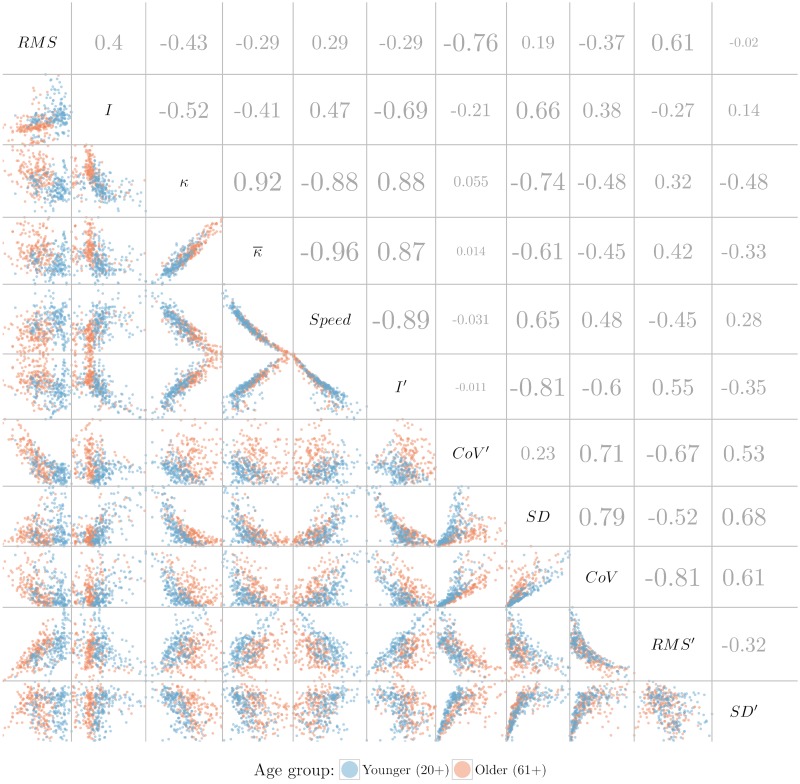
Correlation and scatterplot matrix. Lower triangular matrix: scatterplot matrix, to maintain a good aspect ratio of the plots, values larger than 3.4 from the normalized data (2.5%) were excluded (only for visualization purposes). To estimate the correlation coefficients all data were used. Each point represents a single trajectory. Upper triangular matrix: Pearson correlation matrix. Larger font size indicates stronger correlation, either positive or negative. Absolute correlation values smaller than 0.21 are not significant.

The inherent property of scatter plots to show the relationship between two variables makes a scatter plot matrix an ideal tool to examine the pairwise correlation between multiple variables. In Fig 10, the lower triangular matrix shows the pairwise scatter plots between variables, whereas the upper triangular matrix shows the Pearson correlation coefficients between each pair of measures. A larger font size indicates stronger correlation, either positive or negative. Absolute correlation values smaller than 0.21 are not significant.

Although there are some strong correlations (|*r*| > 0.75) between some measures of dispersion or their variants such as (*SD*,*CoV*) or (*CoV*,*RMS*′), these correlations are not of much interest because this is to be expected given their definitions. We can observe strong positive correlations between *κ* (either mean or median) and *I*′ indicating that higher curvature values are associated with higher turbulence measures. In addition, each corresponding scatter plot shows two clusters, illustrating that younger participants score lower values and older participants score higher values on both measures. We can also observe strong negative correlations between *κ* and *Speed* indicating that higher speed values are associated with lower curvature values and vice-versa.

In addition to the relationships identified in the upper triangular correlation matrix, it is interesting that the lower triangular scatter plot matrix seems to indicate enhanced identification of younger and older clusters in some pairs of variables (see Fig 10), compared to using just a single variable. For example, considering the scatter plot of the pair (*I*′, *CoV*′), we see that young and old separate along a diagonal, meaning that for both measures there are (for example) old participants that are separate from the young participants when considering the combination, but not when considering just one measure. In contrast, the pair (*I*, *CoV*′) shows a mostly vertical separation, indicating that *I* alone is responsible for most of the separation between participants. Since Fig 10 shows quite a few plots with a separation between old and young that is not strictly vertical/horizontal, we expect that further bivariate or multivariate analysis would be promising for the quantification of balance in real-time.

## Discussion

The main goal of this study was to perform a visual data exploration to find measures that can quantify balance continuously during exergaming. In the absence of a gold standard, we considered a measure to be valuable if it showed time-dependent changes in postural control, and differences between older and younger participants in particular. Our visualizations show that *κ*, *Speed* and *I*′ are the most promising measures because (a) they show differences between older and younger participants, (b) their pairwise scatter plots show clusters of younger and older participants, and (c) they can be estimated during game play and they can be used to provide immediate and appropriate feedback.

The heat map and violin plots (Figs 3 and 4) provided qualitative insight into speed and curvature (irregularity) of the CoP trajectories and their variability across younger and older participants. Visualizing balance measures using a heat map showed *Speed* and *κ* differences between older and younger groups, and revealed *I*′ as a valuable measure to differentiate between the two groups. Estimating the overlap between violin plot distributions of young and older adults (Fig 6) provided quantitative measures to select the best variables differentiating older and younger participants (*κ*, *Speed* and *I*′). The parallel coordinate plot provided visual evidence of strong correlation between *κ*, *Speed* and *I*′, and indicated that these measures are not so sensitive to outliers. PCA analysis showed that among other measures *κ*, *Speed* and *I*′ are relevant variables for the first PC. Finally, the correlation and scatter plot matrix illustrated that *κ*, *Speed* and *I*′ might be some of the most promising measures for clustering older and younger participants.

Speed may be a relevant measure of time-dependent balance, as it has been reported to be one of the most reliable age-related measures derived from force plate recordings [68–70]. Speed by itself provides limited information about the quality of movement, as the reasons for a determined speed are unknown. The fact that more irregular trajectories should have higher curvature (*κ*) values can provide additional information on the quality of the movement. To our knowledge, although used in other fields, curvature is a new measure of balance, as it has not yet been used to quantify balance control over time. Thus, *Speed* and *κ* might be two suitable measures for the quantification of balance in dynamic tasks. In addition, the estimation of *κ* and *Speed* does not depend on a reference point like the mean nor on a gold standard, which is a desired feature for a dynamic balance control measure [23]. Turbulence intensity (*I*) is a known measure in fluid dynamics. By the definition of *I*, higher speeds reflect higher *I*-values. Therefore it is natural to expect higher *I*-values for younger than for older participants. However, little is known about its variant (*I*′). Our results show that younger participants score lower *I*′-values than older participants. Although it seems to be a promising measure to differentiate older and younger participants, it not known whether this difference reflects balance control.

Despite showing the best results for clustering older and younger groups, *RMS* and *I*, as indicated in Figs 6 and 10, have two main drawbacks with respect to *Speed* and *κ*: (1) they do depend on a local mean, and (2) it is not clear whether they indicate better or worse balance control. RMS is a traditional measure of balance in static tasks and usually older people show higher RMS variability than younger people. However, RMS is not a common measure for dynamic tasks, and our results actually show higher variability among younger participants. As turbulence intensity (*I*) represents the local variability of *Speed* within a certain time window, the lower *I*-scores among older participants may suggest better postural control (as traditionally interpreted), but this is not expected. Thus, further exploration should clarify the interpretation of these two metrics.

Filtering the data could remove significant variability to differentiate older and younger participants. Thus, it is important to note that we did not smooth the data with a low-pass filter having a certain cut-off frequency between 5 and 15*Hz*, as commonly done for force plate data preprocessing in balance quantification studies [20]. Because our study was exploratory, we used the original lengths of the trials (trajectories), as they may provide additional information about the ability of the participants to play the exergame and to finish the trials. Further research is needed to determine the effect of using different lengths of the trials, as they might have influenced our results to some extent.

Although here we showed visualizations using average and median values, continuous measures can be estimated during real-time performance. Local measures of dispersion and turbulence can be computed for short periods of time, in the order of hundreds of milliseconds. Furthermore, speed and curvature can be computed almost as fast as the data is being recorded, for every two and three samples respectively. In addition to force plates, the measures presented here can be estimated using human motion data recorded by different kinds of tracking technologies such as inertial measurement units and infrared cameras.

Real-time balance quantification during game play could be used to provide immediate feedback to players and to adapt the difficulty level according to their capacity. Appropriate feedback and adaptive game-play in digital exergames could be valuable features to improve motivation to play and to increase their effectiveness as tools to improve balance [15, 71]. In addition, adaptive exergames could increase safety for older adults. As decreasing performance (maybe because of fatigue) could be detected, adaptive exergames could adjust the difficulty level decreasing the risk of falls during exergaming. Conversely, increasing performance of the participant will increase the game-difficulty level that could prevent boredom and abandonment of the game. Another use of balance quantification in real time could be in the field of rehabilitation where improvement could be assessed automatically during exergaming based on the quality of body motions. Finally, a potential and challenging application is the inclusion of this kind of real-time balance control assessment into an expert system to automatically offer advice or personalized medicine to patients with motion impairments.

## Conclusions and future work

Here we have shown how visualization can be used as a way to explore multivariate movement data of young and older adults recorded during exergaming. The properties of heat maps and violin plots can be used to gain quick insight and directions for further exploration. Parallel coordinates, projections, and scatter plot matrices can reveal clusters, patterns, and relationships hidden in the data. Moreover, the creation of such visualizations is straightforward because they are commonly implemented in standard software for statistical analysis.

Even though some single measures show age-related changes, our results suggest that a combination of measures could be more valuable to automatically quantify balance control in real-time, as illustrated by the PCA projection and the pairwise scatterplots.

Possible topics for future research are first to determine the reliability of these measures obtained in home conditions over longer periods of time, using devices that track whole body movements, such as the Kinect. Second, to evaluate the effectiveness of an adaptive exergame as adjusted by the balance measures identified in this study.

## Supporting Information

S1 File Measures of balance. Data (matrix of 400 rows by 11 columns) containing the measures extracted from the trajectories of the center of pressure, and R-code used to generate the figures derived from these the measures.
(ZIP)
